# Deposition of Stainless Steel Thin Films: An Electron Beam Physical Vapour Deposition Approach

**DOI:** 10.3390/ma12040571

**Published:** 2019-02-14

**Authors:** Naser Ali, Joao A. Teixeira, Abdulmajid Addali, Maryam Saeed, Feras Al-Zubi, Ahmad Sedaghat, Husain Bahzad

**Affiliations:** 1 Cranfield University, School of Aerospace, Transport and Manufacturing (SATM), MK430AL Cranfield, England, UK; j.a.amaral.teixeira@cranfield.ac.uk (J.A.T.); a.addali@cranfield.ac.uk (A.A.); 2 Nanotechnology and Advanced Materials Program, Energy and Building Research Center, Kuwait Institute for Scientific Research, Safat 13109, Kuwait; msaeed@kisr.edu.kw (M.S.); fzubi@kisr.edu.kw (F.A.-Z.); 3 Department of Mechanical Engineering, Isfahan University of Technology, Isfahan 84156-83111, Iran; a.sedaghat@ack.edu.kw; 4 Department of Mechanical Engineering, The Australian College of Kuwait, Safat 13015, Kuwait; 5 Imperial College London, Department of Chemical Engineering, SW72AZ London, UK; hb3015@ic.ac.uk

**Keywords:** coating, controlled deposition rate, EB-PVD, morphology, topography, wettability

## Abstract

This study demonstrates an electron beam physical vapour deposition approach as an alternative stainless steel thin films fabrication method with controlled layer thickness and uniform particles distribution capability. The films were fabricated at a range of starting electron beam power percentages of 3–10%, and thickness of 50–150 nm. Surface topography and wettability analysis of the samples were investigated to observe the changes in surface microstructure and the contact angle behaviour of 20 °C to 60 °C deionised waters, of pH 4, pH 7, and pH 9, with the as-prepared surfaces. The results indicated that films fabricated at low controlled deposition rates provided uniform particles distribution and had the closest elemental percentages to stainless steel 316L and that increasing the deposition thickness caused the surface roughness to reduce by 38%. Surface wettability behaviour, in general, showed that the surface hydrophobic nature tends to weaken with the increase in temperature of the three examined fluids.

## 1. Introduction

Stainless steels are passive alloys, which due to their chemical composition tend to form a thin oxide layer that inhibits the metal dissolution in corrosive environments [1]. Physical, mechanical, and anticorrosive properties of the alloy are highly related to its microstructure, where one or two phases (i.e., austenitic, ferritic, or both) may be formed [2]. Due to their unique properties, including adaptation to changes in solution salinity and pH level, these alloys are widely used in application areas such as construction and building [3], heat exchangers [4], and biomedicine [5]. In addition to these application areas, stainless steel (SS) in its powder form was reported to be used in fabricating nanofluids [6,7], which are heat transfer fluids; and surface coatings, via the cold spray deposition method [8]. Since the current trend in engineering industry, such as the automotive sector, is to rely on light construction materials (e.g., aluminium and its alloys) in order to reduce the overall weight of constructions and manufactured parts, hence SS surface coatings on metals are considered to be a promising solution for achieving this target while providing anticorrosion and wear resistance to the bulk material [9,10]. So far, all reported deposition procedures of SS films are seen as adaptation of cold gas dynamic spraying of premixed powders onto the surface [9,11], wire feedstock melt down on surfaces via electron beam solid freeforming (EB-SFF) [12,13], ionic sputtering of a target source [14,15,16,17,18,19,20,21], thermal evaporation of a source and ionic bombardment of the particles by ion beam assisted deposition (IBAD) approach [22], and pulsed laser evaporation technique [23,24]. However, some of these routes can be incompatible for industrial usage because of the lack in precision of controlling the deposited layer thickness, the thin film can be associated with contamination, and the cold spray deposited particles and/or its coated surface can suffer from intensive plastic deformation. Furthermore, the aforementioned methods raise processability and cost concerns due to the large number of parameters involved in the coating procedure. For example, when employing cold gas dynamic spraying approach, parameters such as the nozzle dimensions, jet velocity, particles size, and particles impact temperature need to be considered cautiously before starting the process.

On the other hand, electron beam physical vapour deposition (EB-PVD), which is a high vacuum thermal coating technology, is considered to be a simple and relatively cheap process in which a focused high energy electron beam is directed towards melting an evaporant material inside a vacuumed chamber. The evaporating material is then condensed on the surface of a substrate or component to form the film layer [25]. The distinct advantages of this approach are the high deposition purity, enlarged coating area, precise film thickness, in-situ growth monitoring, and smoothness control [26]. In addition to the associated benefits, the aforementioned technique has proven its capability of depositing alloys, as demonstrated by Almeida et al. [27] with their MCrAlY film fabrication study. On the industrial scale, EB-PVD has been widely employed for coating materials, including SS bulk materials, but to the authors of this article’s knowledge, has never been reported as the means to deposit SS thin films [25,28].

Herein, we demonstrate the deposition of SS thin films on metallic substrates using an EB-PVD approach. The present study, based on the conducted literature review, is the first reported EB-PVD process for forming SS films and does not aim to challenge other film fabrication processes but rather unlocks opportunities for new ways of depositing SS thin films. Furthermore, to illustrate the crucial role of the controlled deposition rates on the uniformity and elemental distribution within the fabricated thin layer, a comparison between the morphologies of the SS films, of 150 nm, coated on copper (Cu) substrates with fixed deposition rates of 0.05 Å/s to 1.45 Å/s was performed. The main reasons behind selecting Cu as the hosting substrate is due to: (1) the fact that Cu is not part of the forming elements of the SS 316L alloy and can therefore be easily diverted from the deposited film when elemental characterisations is performed, and (2) most commercial heat pipes are made of Cu, because of the material high thermal conductivity, but usually faces erosion damages from water flows [29]; so providing an insight into SS coatings on Cu would be desirable for the industry since it can help reduce such common phenomena with minimum degradation effects on the heat transfer properties of the bulk material. Moreover, since there is still a need for further investigation and clarification of the wettability behaviour of SS 316L surfaces and the effect of different parameters on their wetting phenomena [30], the impact of the 0.05 Å/s as-prepared films on the surface topography and water wettability behaviour was explored for 50, 100, and 150 nm SS layers coated on SS 316L substrates. The expected applications that can benefit from this study include, but are not limited to, medical equipment, automotive parts, and heat transfer devices.

## 2. Experimental

### 2.1. Materials

All chemicals were used as-received from the manufacturer without further purification. Acetone (CH₃COCH₃ ≥ 99.5%) grade ACS reagent and hydrochloric acid (HCl ~37%) grade ACS reagent were purchased from SIGMA-ALDRICH, and sodium hydroxide pellets (NaOH ~98%) grade AR were purchased from LOBA Chemie (Mumbai, India). Stainless steel AISI 316L bearing balls, of grade 100 and 8.5 mm diameter, were purchased from Bearing Warehouse Limited (Sheffield, UK). Thirteen square shaped substrates, divided into: (1) four of 3 cm^2^ surface area and 0.6 mm thickness stainless steel 316L (supplied by YC Inox Co., Chang-Hwa, Taiwan), (2) four of 0.5 cm^2^ surface area and 0.6 mm thickness stainless steel 316L (supplied by YC Inox Co.), and (3) five of 1 cm^2^ surface area and 0.127 mm thickness copper of 99.9% purity (supplied by Precision Brand Products, Downers Grove, IL, USA), were manufactured using a computer controlled machine. The substrates were then cleaned with acetone, using a bath type Soniclean limited 250TD ultrasonicator (Thebarton, Australia), for 15 min at room temperature then carefully wiped to remove any remaining residuals. Three litres of deionised water (DIW), of pH 6.11, produced by an Elga PR030BPM1-US Purelab Prima 30 water purification system (Buckinghamshire, UK) was used after being divided into 3 sets of 1 L’s, then their pH value at 25 °C were adjusted, via a HACH HQ11D portable pH meter (Loveland, CO, USA) of 0.002 pH accuracy, to 4, 7, and 9, respectively.

### 2.2. X-ray Fluorescence and X-ray Diffraction Characterisation

Elemental analysis of the fabricated substrates was performed three times and averaged using a BRUKER TITAN S1 X-ray fluorescent (XRF) handheld analyser (Coventry, UK) to ensure that the bulk components in the manufactured substrates match the composition standards. This was done by placing the substrate on a working station then adjusting the lens of the XRF device vertically on the substrate before starting the measurements, which required 10 s to complete for each single measurement. Moreover, X-ray diffraction (XRD) analysis was performed using a 9 kW Rigaku SmartLab, Tokyo, Japan, XRD device that utilizes a CuKα X-ray source at a diffraction angle of 2Ɵ and an incidence beam angle of 0.02°. This was done in order to identify the Bragg’s peaks of each element contained within the substrates and to define the phase of the stainless steel alloy used. The diffraction scanning angle ranged from 20° to 80°, with a scanning rate of 2 °/min.

### 2.3. Stainless Steel Film Deposition

**Figure 1:**
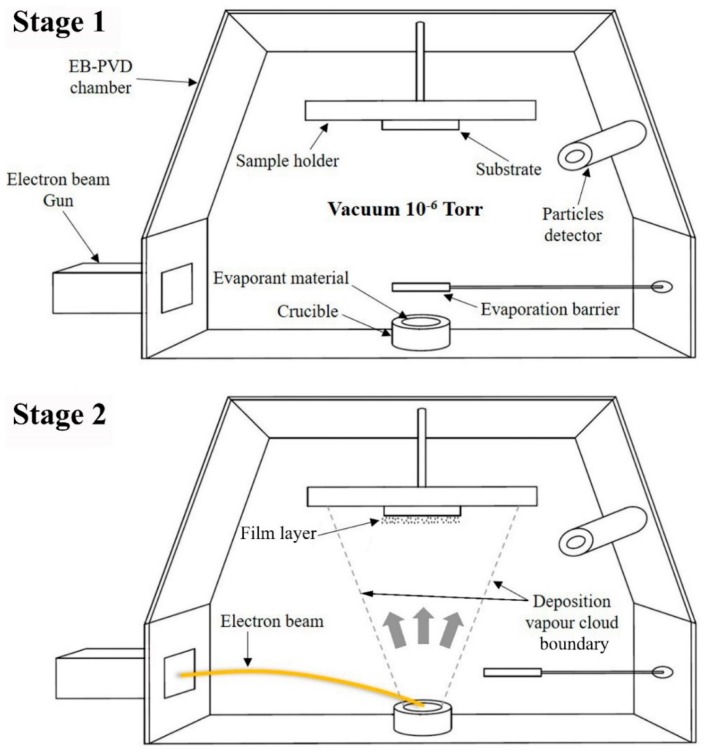
Electron beam physical vapour deposition process, where (Stage 1) shows the schematic illustration of the device configuration, and (Stage 2) demonstrates the source evaporation and film formation process.

The thin film production method used consists of fabrication at constant deposition rates, as commonly seen in literature [31], and is demonstrated in Figure 1. Eleven of the thirteen substrates were individually placed inside the electron beam physical vapour deposition (EB-PVD) device chamber after being tightly adjusted on the sample holder and were screwed vertically above the evaporation source. The remaining two stainless steel 316L substrates were kept as references for characterisation purposes. Stainless steel AISI 316L bearing balls, which were used as the deposition source, were placed in an 8 cm^3^ graphite crucible located at the bottom of the EB-PVD chamber, thus having a fixed target-to-sample distance of 26 cm. The EB-PVD device chamber (40 cm inner diameter × 50 cm inner height) was then vacuumed to a pressure of 6 × 10^−6^ Torr to ensure the removal of all particle contaminations within it, and the film thickness was controlled via an INFICON SQC-310 electronic thickness monitor system (Bad Ragaz, Switzerland) connected to a sensor located inside the chamber. It is worth noting that there was no external heating or cooling applied to the substrates temperature during the film fabrication process. In the case of the copper substrates, the deposition source was evaporated at a set of starting power percentages after which the deposition rates were maintained so that a film layer of 150 nm thick can be achieved. The starting power percentages employed were of 3, 4, 6, 8, and 10%, and the maintained deposition rates were of 0.05, 0.16, 0.82, 1.07, and 1.45 Å/s, respectively. It is worth noting that power percentages less than 3% had no trace of evaporation and that power percentages higher than 12% are restricted by the manufacturer of the EB-PVD device due to safety concerns. As such, the range of power percentage was selected to be from 3% to 10%, with a maximum deviation of ±1% to sustain the deposition rate. As for the stainless steel 316L substrates, based on the elemental characterisation of the film coated on the copper substrates, the evaporation was selected to be at the lowest deposition rate (i.e., 0.05 Å/s) for a set of film thickness of 50, 100, and 150 nm. After the completion of each of the aforementioned particle deposition processes, the substrate was kept in the chamber for 4 h to cool down before removal from the EB-PVD chamber for further analysis.

### 2.4. Scanning Electron Microscopy and Elemental Mapping

The surface microstructure and the elemental mapping of the chemical composition of the 150 nm stainless steel film coated on the copper substrates at fixed deposition rates (i.e., from 0.05 Å/s to 1.45 Å/s); and the evaporated source before and after 150 nm film deposition at a 0.05 Å/s were studied using a JEOL JSM-6010LA InTouchScope^TM^ scanning electron microscopy (SEM, Tokyo, Japan) device that is equipped with an integrated energy dispersive x-ray spectroscopy (EDS) analyser and operates via the InTouchScope 1.12 software. All SEM images were recorded by the secondary electron mode from the surface region of the samples and then recorded at different magnifications. Elemental distribution and percentages were obtained by the EDS analyser at a process real time of 100 s. Both SEM and EDS analysis were conducted at a working distance of 10 mm and an accelerating voltage of 20 kV, to reduce any possible damage to the tested samples. It is important to note that elements such as carbon, oxygen, and copper were excluded from the EDS elemental composition due to the presence of carbon in the adhesive tape used for mounting the samples into the device, traces of oxygen can remain in the chamber even at a high vacuum condition while copper was the tested substrate beneath the thin film, this being our area of interest.

### 2.5. Atomic Force Microscopy

Topography images of the coated and uncoated stainless steel 316L substrates were recorded at room temperature, using a PicoView 1.14.4 software (Woburn, MA, USA), at 10 μm^2^, 1024 × 1024 pixels, and 0.84 line/s via an Agilent Technologies 5600LS atomic force microscopy (AFM, Santa Clara, CA, USA) instrument, equipped with a 90 μm N9521A multipurpose scanner, in tapping mode. NANOSENSORS^TM^ silicon tips (type: PPP-CONTPt-20; resonance frequency 6–21 kHz) were employed for the characterisation. Data analysis was performed, with a Pico Image Basic 6.2 software (Chandler, AZ, USA), via first enabling the gaussian filter with 0.25 μm^2^ cut-off feature, for the background corrections, then having the software calculate the main height parameters and particles height distribution of the samples.

### 2.6. Deionised Water Properties Measurements and Theoretical Calculation

Deionised water, of pH 4, 7, and 9, kinematic viscosity and density changes with temperature were characterised at a temperature range from 20 °C to 60 °C, via an Anton Paar DMA 4500M density meter (Graz, Austria) of accuracy 5 × 10^−5^ g/cm³ and a PAC Herzog HVM 472 multirange viscometer device (Houston, TX, USA), respectively. Both of the aforementioned devices have built-in calibration systems that are initiated before starting the measurements. The variation in pH value, of the DIW’s, with temperature was obtained from previously published results [32,33], for pH 7 and 9, and theoretically calculated for pH 4, for the same range of temperatures, using the following equation [34,35]:

pH_*T*_ = pH_25 °C_ + [(*T* − 25 °C) × solution temperature coefficient] (1)

where pH_*T*_, pH_25 °C_ and *T* are the solution pH value for an examined temperature, the solution pH value at 25 °C, and the examined temperature in Celsius, respectively. The solution temperature coefficient of DIW of pH 4 was selected to be −0.004 pH/°C, based on the extrapolation of the available data of the two previous DIW’s (i.e., pH 7 and 9).

### 2.7. Surface Wettability Characterisation

Three 1 mL Hamilton 1000 series syringes, containing DIW of pH 4, 7, and 9, were adjusted to a Dataphysics SHD syringe temperature controller (Reno, NV, USA), which is integrated with the Dataphysics OCA 100 automatic multi-liquid dispenser contact angle goniometer device (San Jose, CA, USA). This was done in order to increase/decrease the liquid temperature inside the syringes, while being monitored. The surface wettability of the coated and uncoated stainless steel 316L samples was measured using the Sessile drop method [36]. This was done by dispersing a 5 μL droplet (5 μL/s dosing rate) on the examined surface then capturing its image, at static condition, which is analysed afterwards through the device provided software SCA 20 to obtain the liquid – surface CA. The CA measurements were conducted three times, for the three types of liquids, at a fixed liquid temperature of 20 °C to 60 °C, with a ±0.1° contact angle precision.

## 3. Results and Discussion

### 3.1. Substrates Elemental Analysis

**Figure 2:**
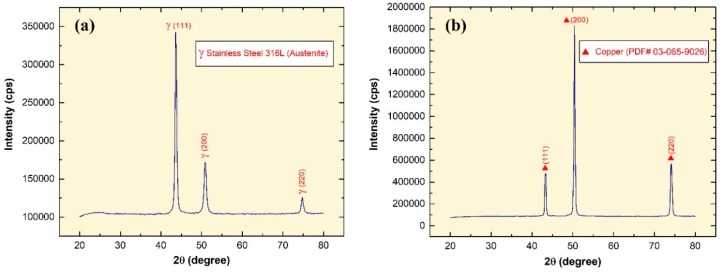
X-ray diffraction pattern of: (**a**) SS 316L substrate, and (**b**) Cu substrate.

The average elemental content of the manufactured SS 316L and Cu substrates, which were each examined three times by the XRF, are given in Table 1. In addition, the XRD pattern corresponds well with the XRF analysis as suggested by the sharp diffraction Bragg’s peaks shown in Figure 2a,b, where the SS substrate (Figure 2a) showed peaks at 2*θ* = 43.6°, 50.9°, and 74.7° corresponding to the planes (111), (200), and (220) austenite gamma phase; and the Cu substrate (Figure 2b) illustrated diffraction peaks at 2*θ* = 43.2°, 50.4°, and 74.1° corresponding to the planes (111), (200), and (220) of pattern (PDF Card No.: 03-065-9026). Hence, this confirms that the bulk formation of the as-prepared substrates used for the deposition experiments are of Cu and SS 316L in its austenite phase.

### 3.2. Deposition Morphology

**Figure 3:**
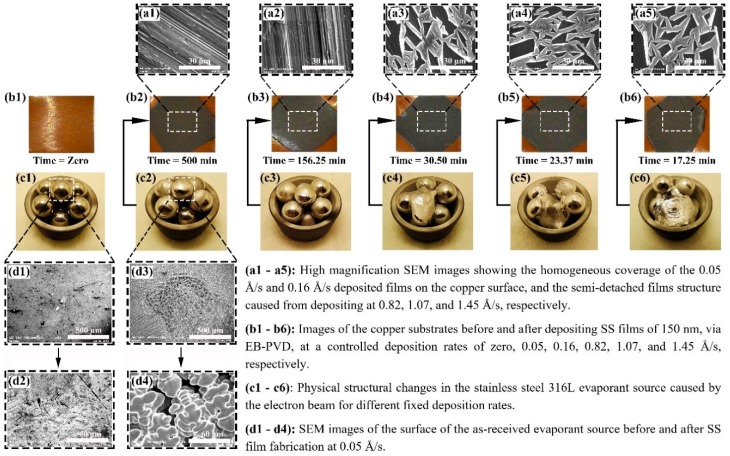
Characterisation of the Stainless steel evaporant source and deposited thin films, where (**a1**–**a5**) shows the SEM images of the 0.05–1.45 Å/s as-deposited films structure, (**b1**–**b6**) illustrates the copper substrates before and after SS deposition, (**c1**–**c6**) demonstrates the physical changes in evaporant source caused by different deposition rates, and (**d1**–**d4**) shows the SEM images of the evaporant source before and after 0.05 Å/s film deposition.

**Figure 4:**
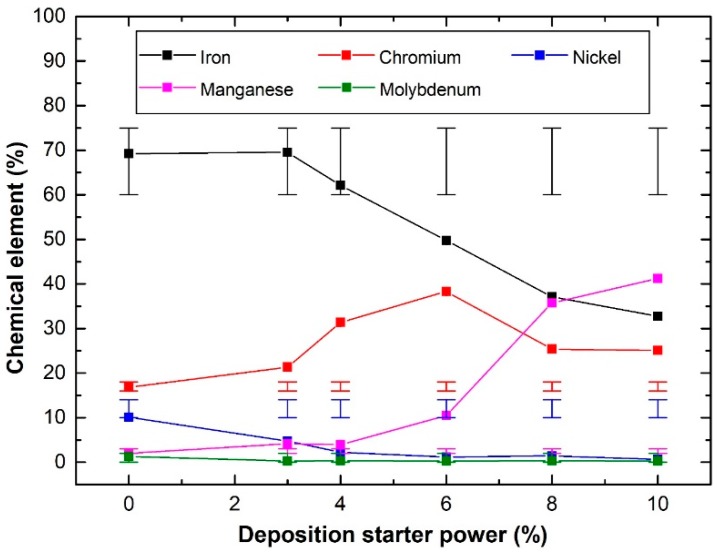
EDS characterisation of the chemical elemental percentages of the evaporant source (0% power), and the SS thin films deposited with a starting beam power of 3% up to 10%. The bars at the top and bottom of each data point indicate the maximum and minimum range of each element percentage of the stainless steel 316L composition and was attained from the XRF device installed database.

**Figure 5:**
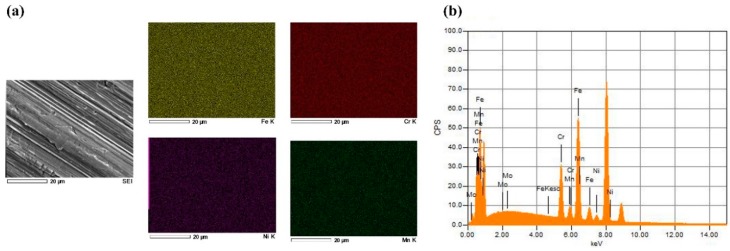
EDS elemental analysis, where (**a**) is the SEM image and its elemental maps of the characterised 150 nm deposited SS film at 0.05 Å/s on Cu substrate, and (**b**) demonstrates the EDS X-ray spectrum of the elements within the film shown in (**a**).

Fabricated samples of 150 nm SS deposited on Cu substrates, which were synthesised by the EB-PVD starting electron beam powers of 3% to 10%, are shown in Figure 3. The SS thin film layer produced can be visually observed (Figure 3(b2–b6)), except for the corners where the adhesive tapes were placed to adjust the samples on the sample holder. The time required for achieving the film thickness varied from 500 min, at a fixed deposition rate of 0.05 Å/s, down to 17.25 min, via a 1.45 Å/s controlled evaporation rate. Samples were then characterised to determine the effect of the controlled deposition rates on the film and evaporant material (Figure 3(c1–c6)) morphologies, via the SEM and EDS analysis. The SEM characterisation of the 0.05 Å/s and 0.16 Å/s fabricated samples has demonstrated a well-constructed film structure as shown in Figure 3(a1,a2) (alternatively, Supplementary Figure S1(a1,a2)), whereas higher deposition rates have resulted in the development of non-uniform films with partial detachments from the surface as seen in Figure 3(a3–a5) (alternatively, Supplementary Figure S1(a3–a5)). Both observations can be linked to the phase of the SS film formed due to the elemental ratio of the deposit, where a well-constructed film structure indicates a ferritic phase and a semi-detached structure illustrates an austenitic phase [37]. In addition, analysing the evaporant source, before and after 0.05 Å/s deposition, has shown a local melt down in the material, due to the electron beam being focused on a single location at a low power (i.e., the beam is fixed and does not follow a movement path across the evaporant source), thus resulting in surface microstructural changes as shown in Figure 3(d1–d4) (alternatively, Supplementary Figure S1(b1–b4)). Further inspection of the samples using the EDS device (Figure 4) has illustrated that, unlike higher controlled deposit rates, the film fabricated via 0.05 Å/s was within the SS 316L elemental composition acceptable ranges, except for the chromium, manganese, and nickel, which showed partial divergences of +3.33%, +1.15%, and −5.24%, respectively. This indicates that the film is indeed SS but of a different category [38,39,40], whereas to obtain SS 316L thin films the evaporant source would require some modification in its composition (i.e., fabricate our own EB-PVD evaporant source). In addition, the film elemental distribution of the fixed 0.05 Å/s deposition was seen to be uniformly distributed along the substrate surface as shown in Figure 5 (alternatively, Supplementary Figure S2a–b). It is important to note that due to the limitation of the EDS device used, elements of less than 0.5% of the composition mass could not be elementally mapped, as seen in the case of molybdenum. The EDS elemental composition percentages of the 150 nm SS deposited film at a fixed rate of 0.05 Å/s are tabulated in Table 2.

### 3.3. Surface Topography

**Figure 6:**
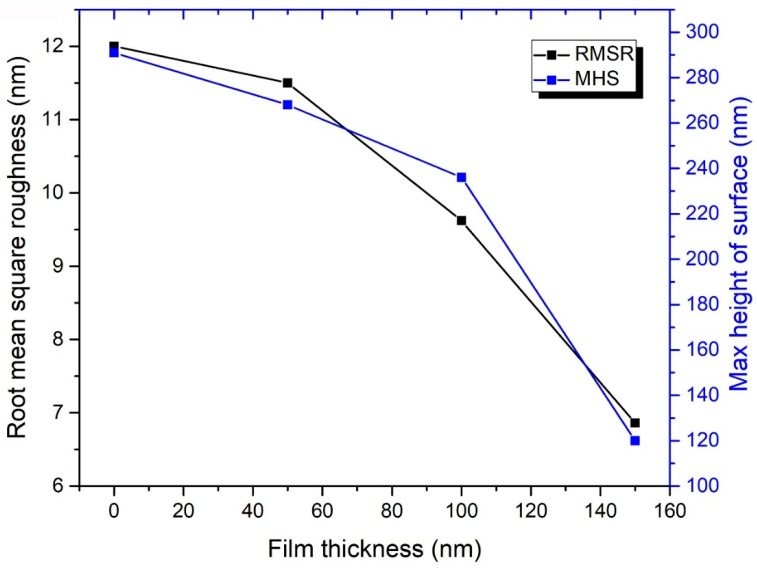
Root mean square roughness and maximum height of surface variation with deposition thickness on SS 316L substrates.

**Figure 7:**
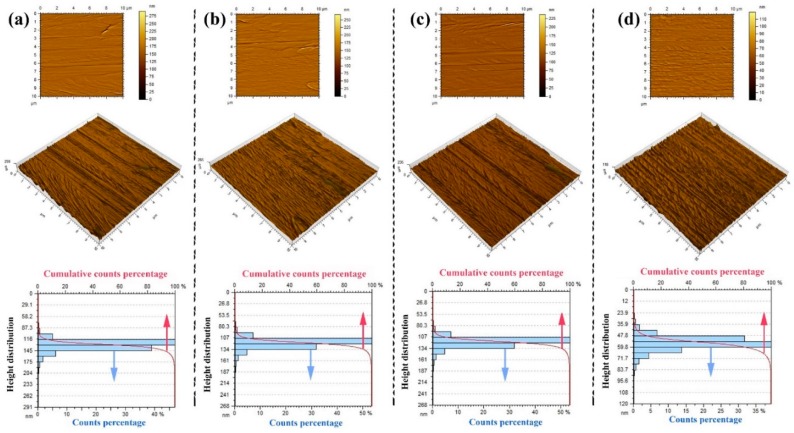
Surface topography analysis of SS films on SS 316L substrates, where (**a**) 2D and 3D rendered AFM topograph and height distribution of the surface of the uncoated SS 316L substrate, and (**b**–**d**) 2D and 3D rendered AFM topograph after 50, 100, and 150 nm SS deposition on substrates and their height distribution.

Surface topography analysis of the uncoated and coated SS 316L samples, which were conducted using an AFM device, is shown in Figure 6 and Figure 7 (alternatively, Figure 3a–d). The experimental results have revealed that the nanostructures on the uncoated surface have a range of height between 87.3 to 204 nm (Figure 7a), with almost 47.5% of the structure height being in the range of 116 to 130.5 nm. Moreover, the maximum height of surface (MHS), which was obtained by adding up the surface maximum peak height and maximum valley depth, and root mean square roughness (RMSR) of the uncoated sample, were shown to be 291 nm and 12 nm, correspondingly, as demonstrated in Figure 6. On the other hand, as the deposited layer thickness increased (Figure 7b–d), the structure height on the surface, RMSR, and MHS were seen to reduce, reaching values between 35.9 to 83.7 nm, 6.86 nm, and 120 nm, respectively. Furthermore, the degree of symmetry of the surface heights about the mean plane was also seen to improve with the deposited film thickness, as the obtained skewness (Ssk) values of the measured samples (Table 1) were shown to move closer to the mean plane (i.e., zero) with the increase in fabricated layer thickness. It is worth noting that the sign of Ssk represents the predominance of the comprising surface peaks (Ssk > 0) or valley structures (Ssk < 0). The aforementioned changes in surface conditions can be attributed to the deposited particles occupying the vacant spaces on the surface structure, which consist of valleys, hills, and micro gaps, leading to the height variation on the surface to narrow down [41]. Moreover, the presence of inordinately high peaks or deep valleys was also found on the examined substrates, as indicated by the surface kurtosis (Sku) values, where Sku > 3.00 suggests the existence of a peaks/valleys defect on the surface and Sku < 3.00 illustrates a lack thereof (i.e., insufficient surface information). Such an observation is not surprising as it is commonly present on most surfaces [42]. The average roughness values were found to be 7.87 nm, 7.48 nm, 6.00 nm, and 4.88 nm for the uncoated, 50 nm, 100 nm, and 150 nm coated substrates, correspondingly. These results confirmed the smoothening effect caused by the increase in EB-PVD deposited film thickness on the SS 316L substrate surface. The roughness results can also be used as a general indication of the corrosion behaviour, as it has been reported by other authors that decreasing the surface roughness of passive alloys tends to reduce the pitting susceptibility and corrosion rate [43,44]. The height parameters values obtained from the AFM analysis of the samples can be seen in Table 3.

### 3.4. Deionised Water Properties Variation with Temperature

**Figure 8:**
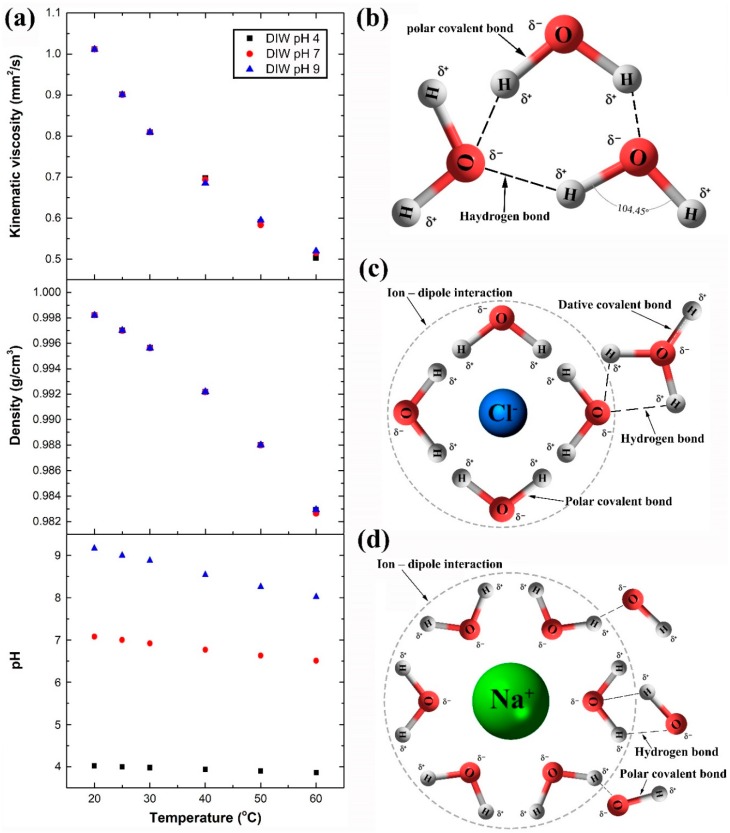
Water atoms and molecules bonds, and properties variation with temperature, where (**a**) shows the DIW’s kinematic viscosity, density, and pH value changes with temperature, and (**b**–**d**) illustrates the bonds in water of pH 7, 4, and 9, respectively.

The deionised waters used were selected to have a pH of 4, 7, and 9 to observe the acidity, neutrality, and alkalinity of the liquid effect on the surfaces wettability. Analyses results of the changes in properties, namely, kinematic viscosity (ν), density (ρ), and pH value of the three liquids, within our temperature range, are shown in Figure 8a. Comparing the ρ and ν characterisation outcomes, of the as-prepared DIW of pH 7, with the available data on pure water in literature [45] has shown a deviation of 0.015% and 3.67%, respectively, thus verifying the measurement approach conducted. Moreover, regardless of the examined DIW pH value, the as-fabricated liquids ν and ρ were seen to have a negligible difference in their values at each investigated point of temperature. For example, at 30 °C, the DIW $\rho_{\mathrm{pH}4}$, $\rho_{\mathrm{pH}7}$ and $\rho_{\mathrm{pH}9}$ ad an outcome of 0.99564, 0.99562, and 0.99563 g/cm^3^, respectively. In contrast, manipulating the temperature was seen to have a notable influence on all three properties of the DIW’s (i.e., ν, ρ, and pH value), as demonstrated in Figure 8a. This can be explained by the fact that ν is inversely related to the ρ, and that ρ is a representation of substance mass to its volume, where at a constant volume, the mass is influenced by the bonds distance of the molecules and their forming atoms. Our as-prepared DIW’s consist of four types of bonds: (1) Polar covalent bond between a single or a pair of hydrogen atoms and one atom of oxygen, (2) Dative covalent bond between a single atom of H^+^ and a H_2_O molecule, (3) hydrogen bond between the oxygen atom of a H_2_O molecule and a hydrogen atom of a neighbouring H_2_O molecule, and (4) Ion–dipole interaction between the H_2_O molecules and a Cl^−^ atom (e.g., DIW of pH 4) or Na^+^ atom (e.g., DIW of pH 9). The four previous bounds are shown in Figure 8b–d. Based on the obtained data, it is believed that at a point of temperature, the bond distances of the newly introduced dative covalent bond (pH 4) and ion – dipole interaction (pH 9) are very close in distance to the other two initially existing bonds in neutral water, causing this neglectable changes in the liquid mass. On the other hand, raising the temperature weakens all four bonds, because of the increase in molecular vibrations, causing the bonds distance to widen; and hence the liquid mass reduces and becomes more acidic due to the release of H^+^ and growth in its concentration [46]. The different formed reactions in our as-prepared DIW’s, based on the Bronsted–Lowry theory of acids and bases [47], are demonstrated in Equations (2)–(4) as the following:

pH 4: HCl + H_2_O ⇌ H_3_O^+^ + Cl^−^ (Reverse reaction) (2)

pH 7: 2H_2_^+^+ O_2_^−^ → 2H_2_O (Chemical reaction) (3)

pH 9: NaOH + H_2_O → Na^+^ + OH^−^ + H_2_O + Heat (Exothermic reaction) (4)

### 3.5. Contact Angle Measurement

**Figure 9:**
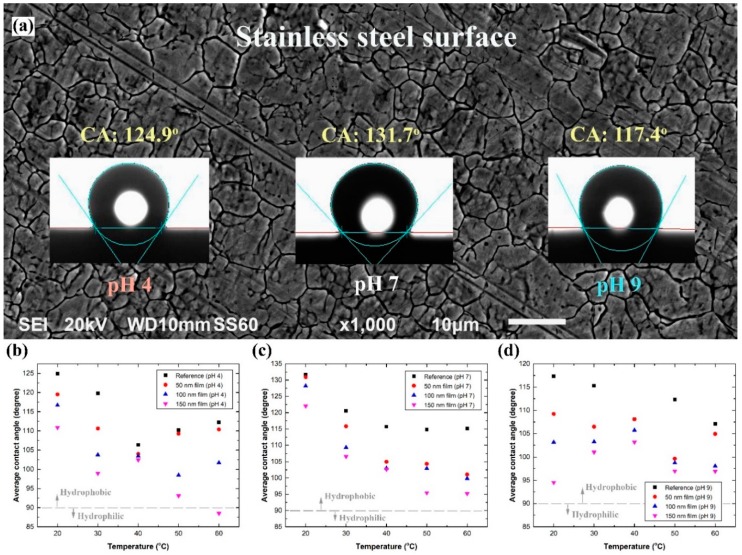
Effect of DIW temperature and pH value on the wettability behaviour of SS 316L surface, where (**a**) illustrates the contact angles between the 20 °C DIW’s, of pH 4, 7, and 9, and the uncoated SS 316L substrate surface, and (**b**–**d**) demonstrates the average contact angle measurements of the uncoated and coated samples using the DIW’s, at 20–60 °C, as the testing fluids.

Examining the wettability of the uncoated SS 316L sample (Figure 9a), with 20 °C of DIW of pH 7, showed that the surface had an average contact angle (ACA) of 131.7°, which illustrates a hydrophobic behaviour. The high ACA value is believed to be linked to the substrate Cassie–Baxter state via its surface roughness, as reported by other authors [30,48]. In general, there are two common texture states that explain the relationship between surface wettability and roughness, which are the Cassie-Baxter [49] and Wenzel [50] states. In the Cassie-Baxter state, the surface pores and valleys tend to trap the air, which leads to a reduction in the degree of liquid-surface interaction. On the other hand, in the Wenzel state, the liquid fully occupies the pores, thus improving the surface wettability. Applying DIW’s of pH 4 and 9 have led the surface ACA to reduce to 124.9° and 117.4°, respectively, without additional surface modifications. Furthermore, it was found that raising the temperature of the as-prepared DIW’s tends to weaken the hydrophobic nature of the uncoated surface, as demonstrated by the obtained data in Figure 9b–d. The grey dashed line in the plots (Figure 9b-d) illustrates the transition point between the surface hydrophobic (top) and hydrophilic (bottom) regions. Moreover, the deposition thickness was seen to be inversely related to the CA, where increasing the film thickness caused the ACA to reduce. For example, when examining DIW, of 20 °C and pH of 4, the ACA of the uncoated, 50 nm, 100 nm, and 150 nm film gave angles of 124.9°, 119.5°, 116.7°, and 110.9°, correspondingly. This can be attributed to the reduction in surface micro-roughness and air pockets formation at the interface between the substrate and liquid as a result of the deposited particles occupying the surface structure, thus enhancing the substrate surface energy to attract the liquid towards the surface (i.e., reducing the CA) [49,51]. In addition, the level of decrease in CA is seen to correspond to the liquid temperature, pH value, and fabricated film thickness due to their ability to modify the surface mode from a Cassie–Baxter state to a Wenzel state, and vice versa. For instance, the ACA of DIW of pH 4, 7, and 9, at 50 °C, showed a decrease from 110.1°, 114.8°, and 112.3° (uncoated) to 93.1°, 95.5°, and 97.0° (150 nm film), respectively. It was also possible to change the substrate surface wettability nature from hydrophobic to hydrophilic by manipulating the three aforementioned parameters, as shown in Figure 9b when investigating the 150 nm coated substrate with DIW of 60 °C and pH 4. Such findings are very attractive for heat transfer applications, as lowering the CA can enhance the heat transfer efficiency of SS by providing larger contact area between the liquid and the surface. On the other hand, the fluctuation in the data trend of DIW of pH 4 and 9 across the examined temperature range is believed to be caused by the free ions hosted by the liquid. Since both fluids are considered ionically unstable, attempting to change the surface functional group of the substrate from hydrophobic toward hydrophilic by inducing the transformation in surface charge, in what is known as the Hofmeister series reversal effect, could be the reason behind this kind of behaviour in the data trend [52]. In addition to the previously mentioned reason, there is a possibility that traces of hydrocarbon contamination from the surrounding atmosphere on the substrate surface are strongly interacting with the two aforementioned liquids free ions, since the DIW of pH 7 did not show such fluctuation in its data trend. Usually, such contamination is unavoidable in open atmospheric experiments, and would have some sort of influence on all the conducted measurements [53,54]. Supplementary Table S1 summarises the testing parameters and obtained contact angles of the characterised samples.

## 4. Conclusions

Stainless steel films were fabricated via an electron beam physical vapour deposition method with starting electron beam power percentages of 3%–10%. The thin layers obtained with a controlled deposition rates of 0.05 Å/s and 0.16 Å/s have shown a uniform elemental distribution with a well-constructed film structure that covered the whole exposed area, while higher deposition rates have illustrated semi-detachments in the film structure. Furthermore, the closest film elemental content to SS 316L was achieved with a 0.05 Å/s, where higher deposition rates were seen to extend the maximum and minimum elemental limits of SS 316L. Surface topography of SS 316L before and after depositing 50 nm, 100 nm, and 150 nm films, using controlled 0.05 Å/s fabrication rate, was then examined. The results illustrated a reduction in structure height on the surface, MHS, RMSR, and average roughness from 87.3–204 nm, 291 nm, 12 nm, and 7.87 nm (uncoated substrate) to 35.9–83.7 nm, 120 nm, 6.86 nm, and 4.88 nm (150 nm coated substrate), respectively. It also showed, via the obtained Ssk values, that the degree of symmetry of the surface heights about the mean plane was improved by ~49.5% for the reference substrate after 150 nm film deposition. Surface wettability of the as-prepared samples were afterwards characterised with DIW’s, of pH 4, 7, and 9, at a 20–60 °C liquid temperatures. The film thickness was seen to be inversely related to the liquid–surface CA, and hence the CA reduced with the increase in film thickness. Moreover, the rise in DIW’s temperature has been shown to weaken the hydrophobic nature of the as-prepared substrates. It was also noticed that, unlike the DIW of pH 7, the liquids of pH 4 and 9 demonstrated some fluctuation in their CA data trend.

In summary, this article unlocks a new approach for depositing stainless steel thin films using an electron beam physical vapour deposition technique. The resulting film is ultrathin, uniform, conformal, and controllable. Moreover, an extension towards depositing different grades of stainless steel can be achieved by changing the composition of the evaporant source, based on exploratory experiments; and hence may facilitate a feasible route towards industrial usage of the process after further film properties investigation is provided (e.g., corrosivity, cohesion, hardness, and abrasiveness). Furthermore, as our approach is the first example of any stainless steel EB-PVD coating, the present work marks an important milestone in the future of stainless steel depositions on metallic surfaces and is expected to be beneficial to many applications such as medical equipment, automotive parts, and heat transfer devices.

## Figures and Tables

**Table 1:** Averaged XRF elemental analysis of SS 316L and Cu substrates.

Element	Stainless Steel 316L				Element	Copper			
	Content	Max.	Min.	+/− Error		Content	Max.	Min.	+/− Error
	(%)	(%)	(%)	(%)		(%)	(%)	(%)	(%)
Iron	69.22	75	60	0.5	Copper	99.82	100	90	0.39
Chromium	16.78	18	16	0.18	Zirconium	0.02	-	-	0.01
Nickel	10.12	14	10	0.21	-	-	-	-	-
Manganese	2.02	3	2	0.04	-	-	-	-	-
Molybdenum	1.32	2	0	0.11	-	-	-	-	-
Silicon	0.27	1	0	0.05	-	-	-	-	-

**Table 2:** EDS elemental composition percentage of the 150 nm SS fabricated film at 0.05 Å/s controlled deposition rate.

Element	Mass %	Atom %	Sigma	Net	K ratio
Iron	69.52	68.62	0.05	2879132	0.3919295
Chromium	21.33	22.61	0.03	1347161	0.1368063
Nickel	4.76	4.47	0.03	131065	0.0250911
Manganese	4.15	4.16	0.03	201285	0.0236898
Molybdenum	0.25	0.14	0.03	12922	0.0009314
Total	100	100	-	-	-

**Table 3:** Height parameters of the AFM analysis of the uncoated, 50 nm, 100 nm, and 150 nm coated SS substrates.

Height Parameters	Film Thickness (nm)			
	0	50	100	150
Root mean square roughness (nm)	12	11.5	9.62	6.86
Skewness	−1.08	−1.0	−0.654	−0.535
Kurtosis	16.8	18	19.6	6.39
Maximum peak height (nm)	131	119	119	55.4
Maximum valley depth (nm)	160	149	118	64.1
Maximum height of surface (nm)	291	268	236	120
Average roughness (nm)	7.87	7.48	6.0	4.88

## Supplementary Materials

The following are available online at http://www.mdpi.com/1996-1944/12/4/571/s1, Figure S1: (a1) SEM image of the 0.05 Å/s deposited film; (a2) SEM image of the 0.16 Å/s deposited film; (a3) SEM image of the 0.82 Å/s deposited film; (a4) SEM image of the 1.07 Å/s deposited film; (a5) SEM image of the 1.45 Å/s deposited film; (b1) SEM image of the as-received evaporant source before film deposition; (b2) Higher resolution SEM image of the as-received evaporant source before film deposition; (b3) SEM image of the as-received evaporant source after 0.05 Å/s film deposition; (b4) Higher resolution SEM image of the as-received evaporant source after 0.05 Å/s film deposition; Figure S2: (a) SEM image and its elemental maps of the characterized 150 nm deposited SS film at 0.05 Å/s on Cu substrate; (b) EDS X-ray spectrum of the elements, Figure S3: (a) AFM images and analysis of the uncoated substrate; (b) AFM images and analysis of the 50 nm coated substrate; (c) AFM images and analysis of the 100 nm coated substrate; (d) AFM images and analysis of the 150 nm coated substrate, Table S1: Contact angle measurements data.
