# Supplementary material for: Deposition of Stainless Steel Thin Films: An Electron Beam Physical Vapour Deposition Approach
Source: Materials (Basel). 2019 Feb 14;12(4):571. doi: 10.3390/ma12040571 (PMC6416557; doi:10.3390/ma12040571)
Supplement: Supplementary file 1 [file materials-12-00571-s001.pdf]

materials

Supplementary materials: Deposition of Stainless Steel Thin Films: An Electron Beam Physical Vapour Deposition Approach

Naser Ali, Joao A. Teixeira, Abdulmajid Addali, Maryam Saeed, Feras Al-Zubi, Ahmad Sedaghat and Husain Bahzad

SEM images of the Stainless steel evaporant source and deposited thin films:

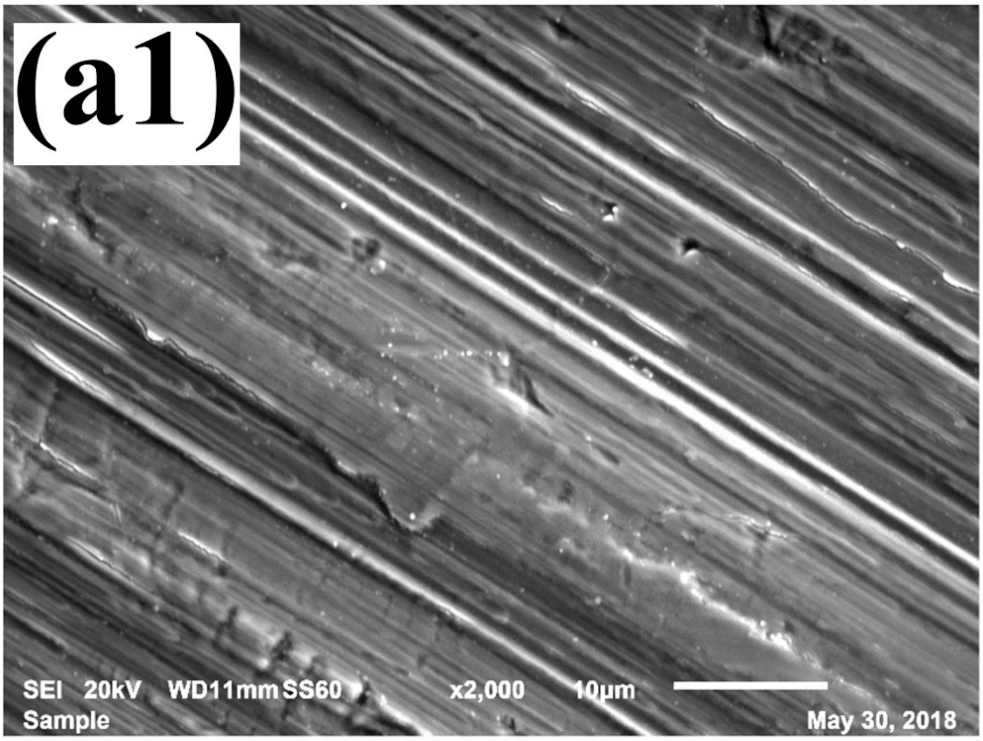

Figure S1. (**a1**) SEM image of the 0.05 Å/s deposited film.

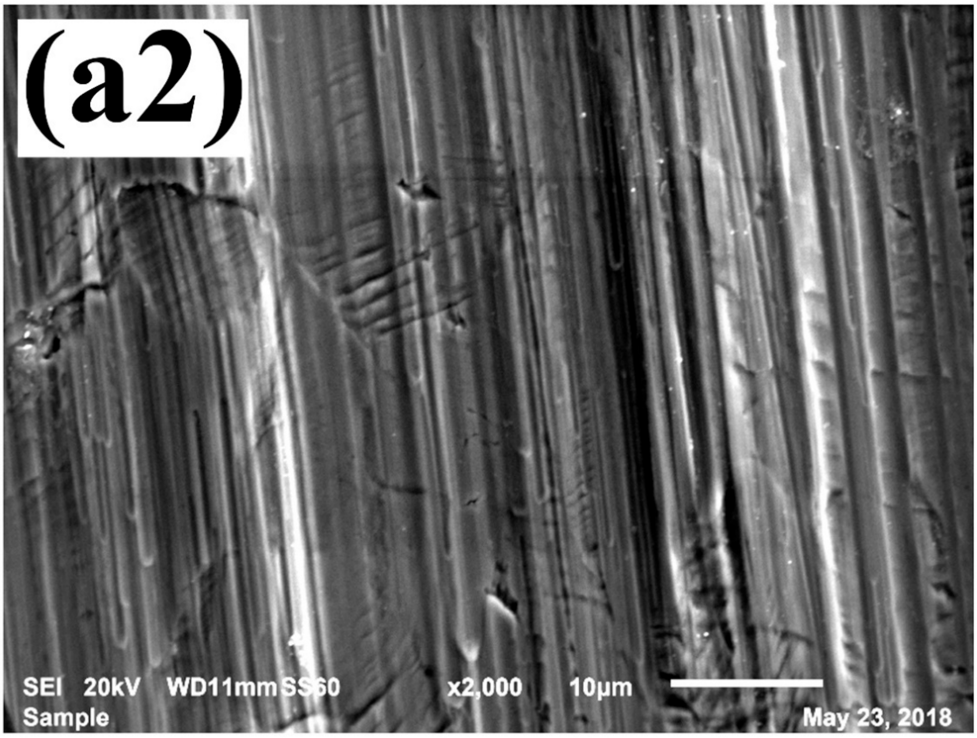

Figure S1. (**a2**) SEM image of the 0.16 Å/s deposited film.

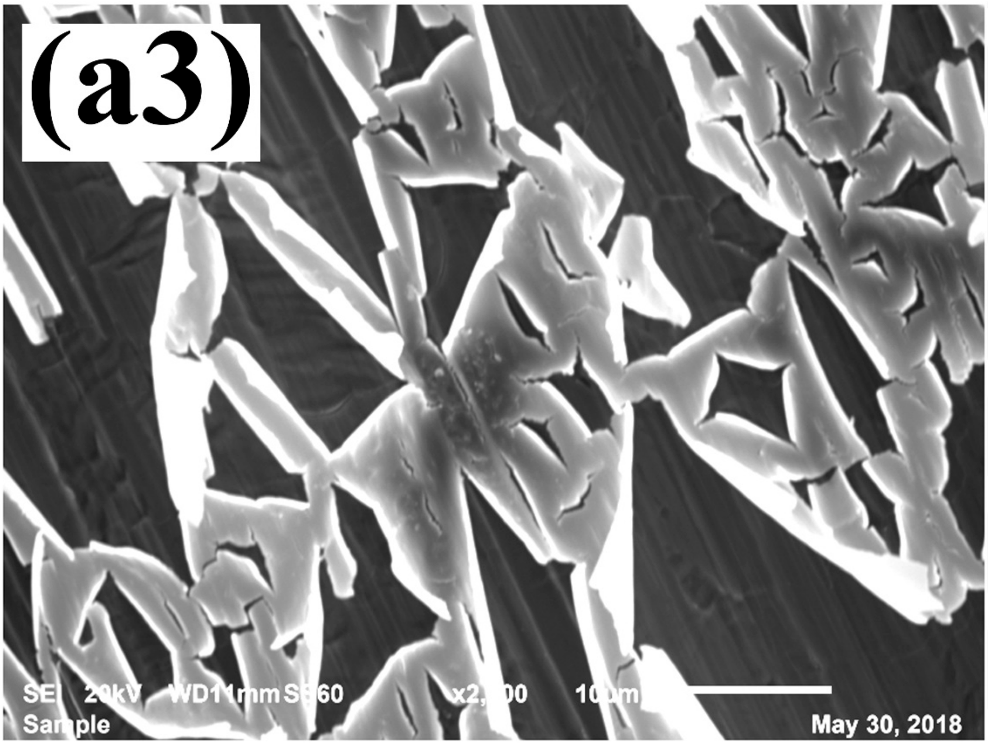

Figure S1. (**a3**) SEM image of the 0.82 Å/s deposited film.

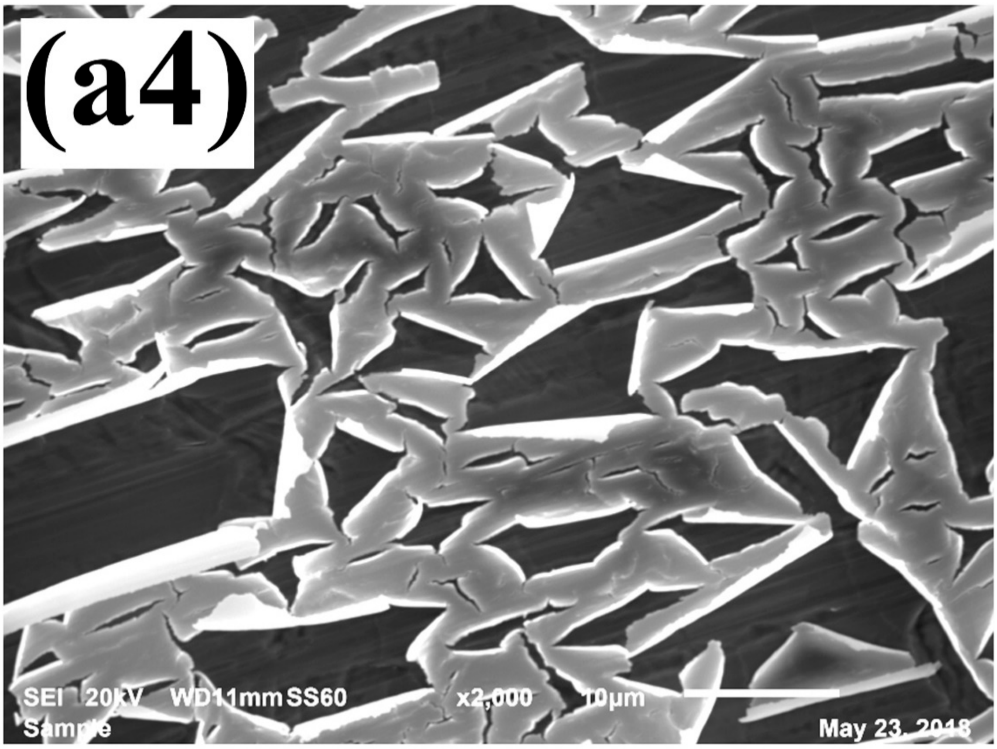

Figure S1. (**a4**) SEM image of the 1.07 Å/s deposited film.

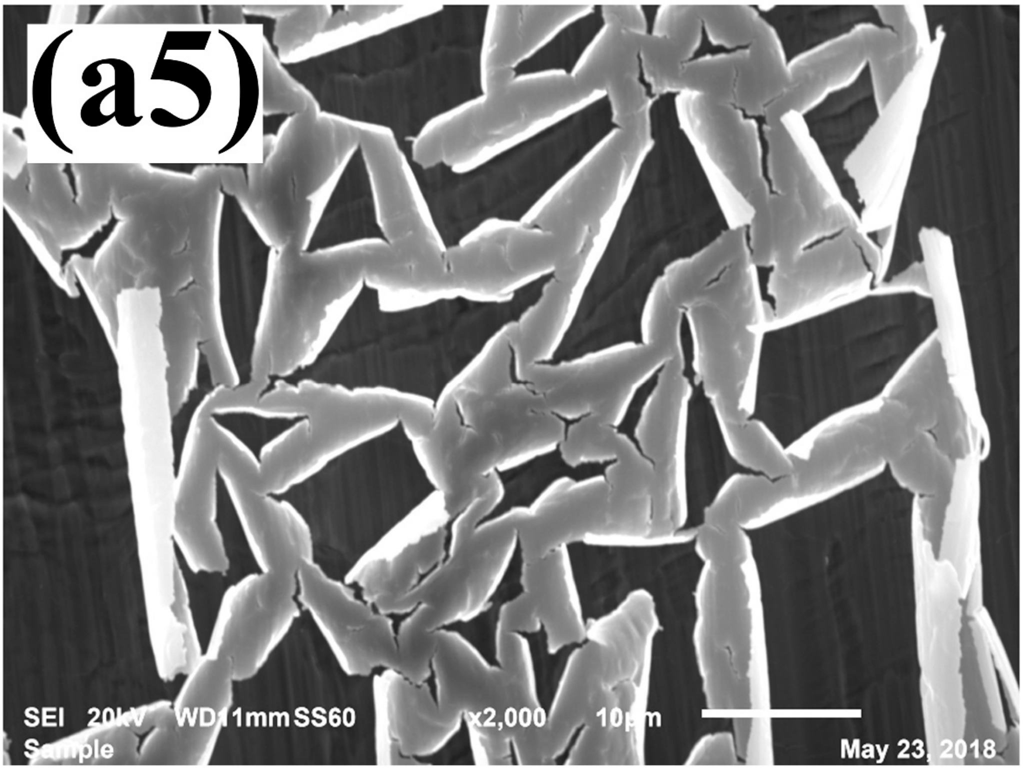

Figure S1. (**a5**) SEM image of the 1.45 Å/s deposited film.

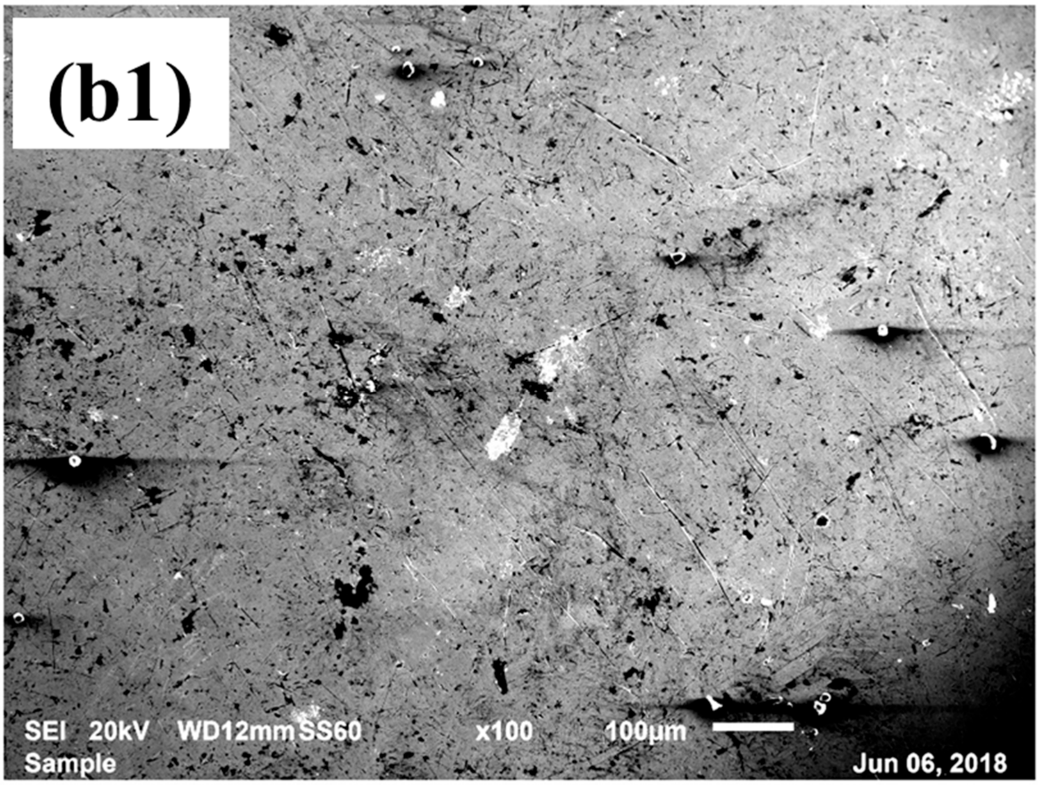

Figure S1. (**b1**) SEM image of the as-received evaporant source before film deposition.

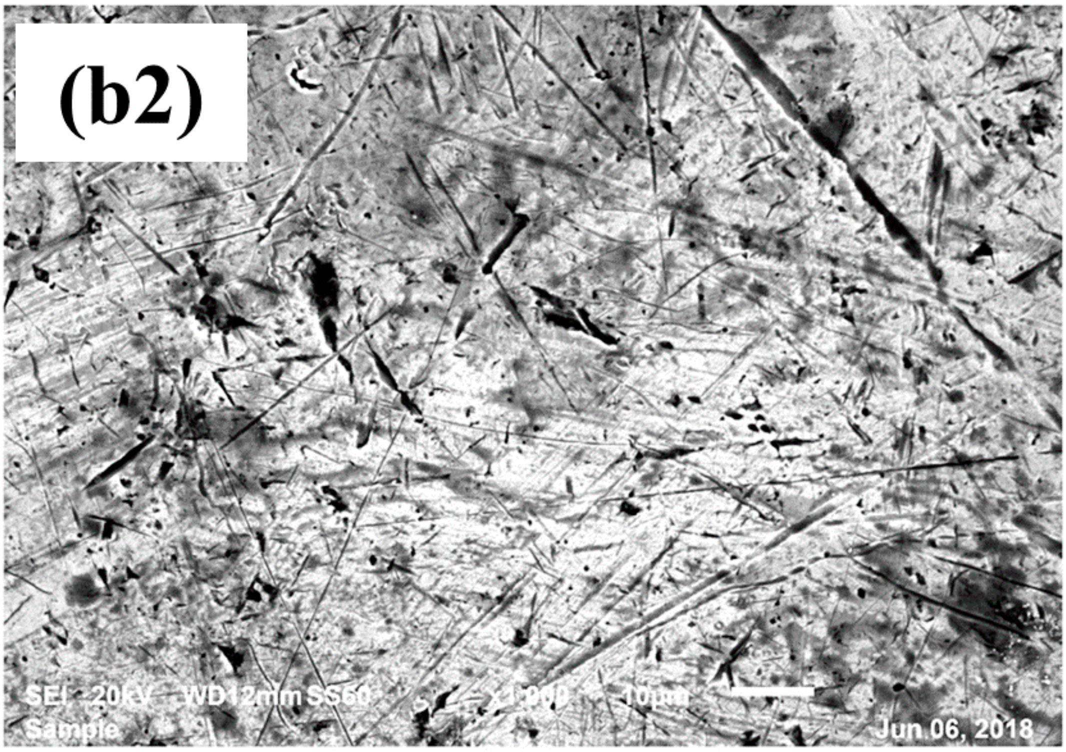

Figure S1. (**b2**) Higher resolution SEM image of the as-received evaporant source before film deposition.

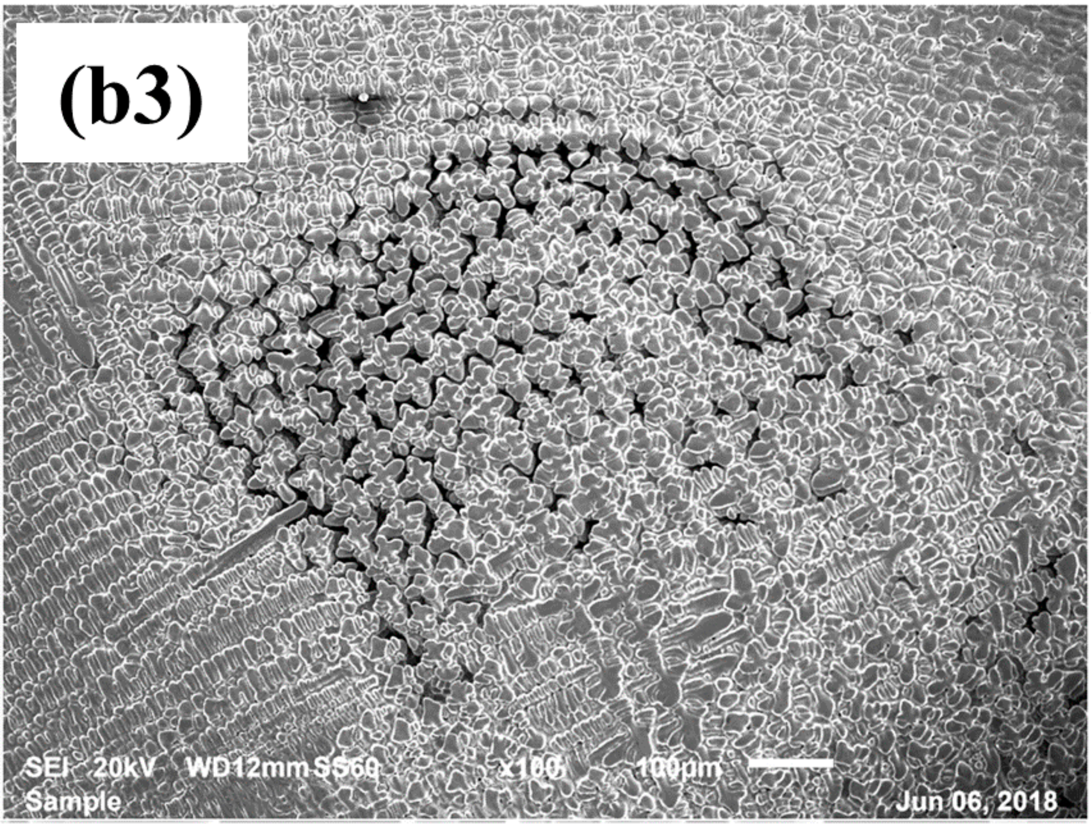

Figure S1. (**b3**) SEM image of the as-received evaporant source after 0.05 Å/s film deposition.

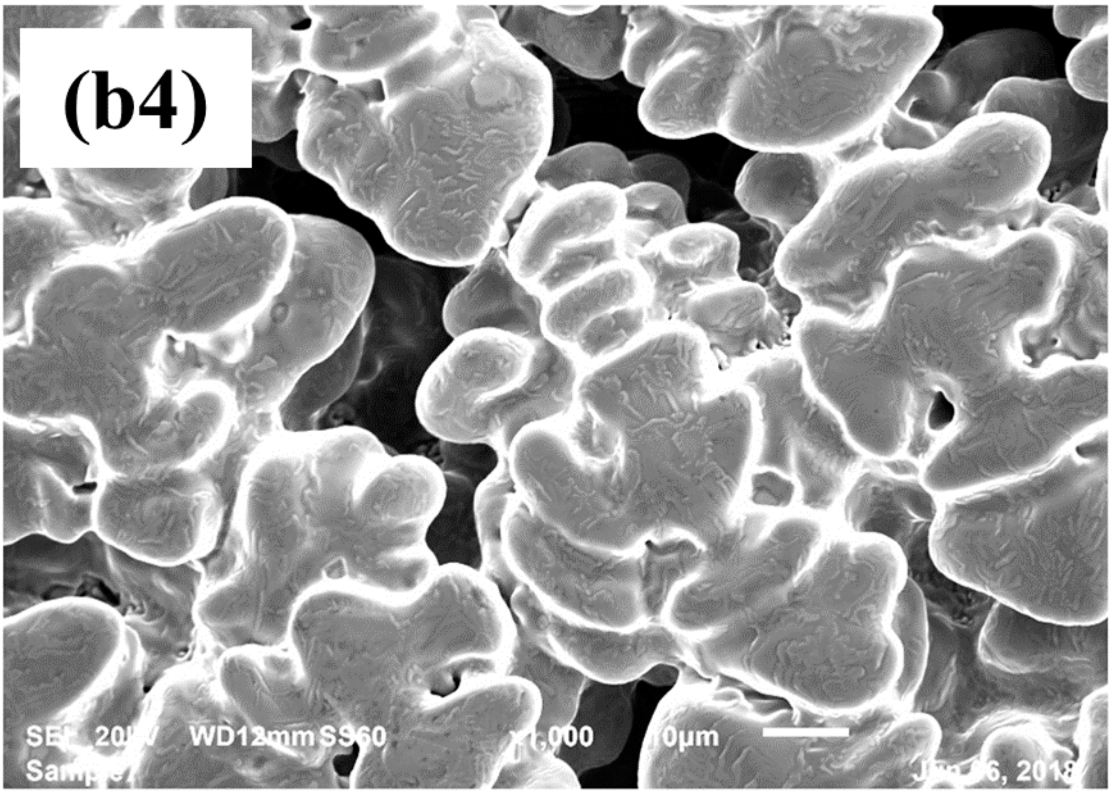

Figure S1. (**b4**) Higher resolution SEM image of the as-received evaporant source after 0.05 Å/s film deposition.

Deposited film EDS elemental analysis:

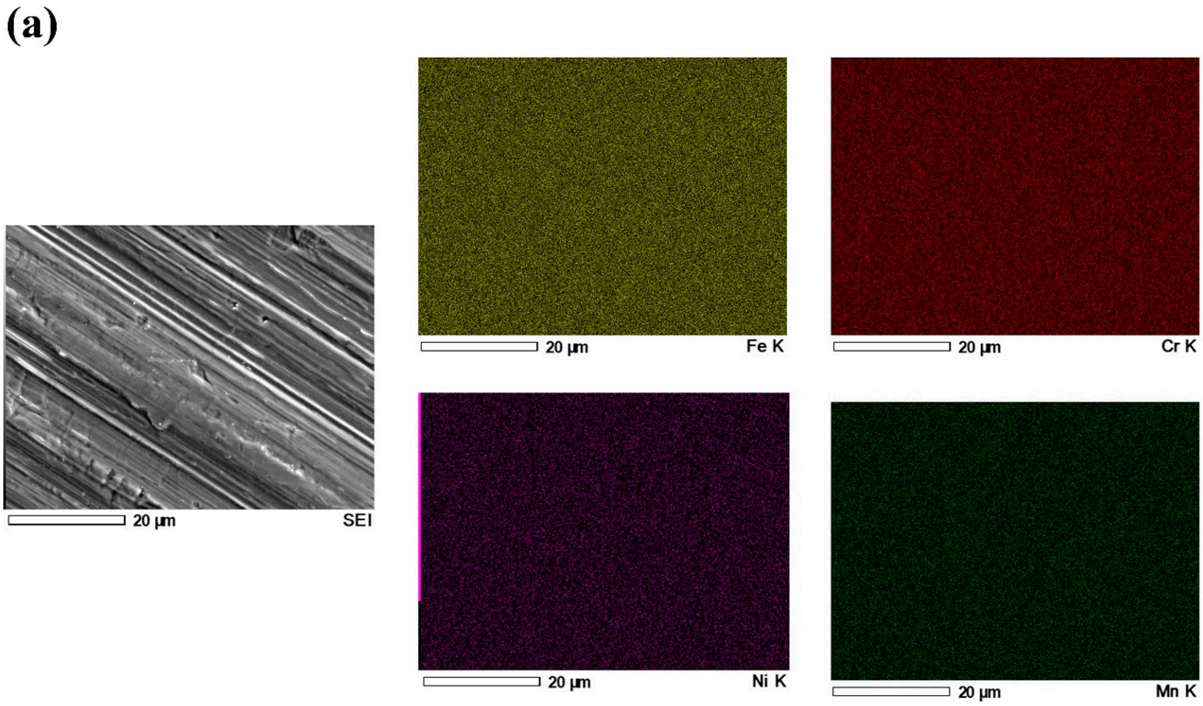

Figure S2. (**a**) SEM image and its elemental maps of the characterized 150 nm deposited SS film at 0.05 Å/s on Cu substrate.

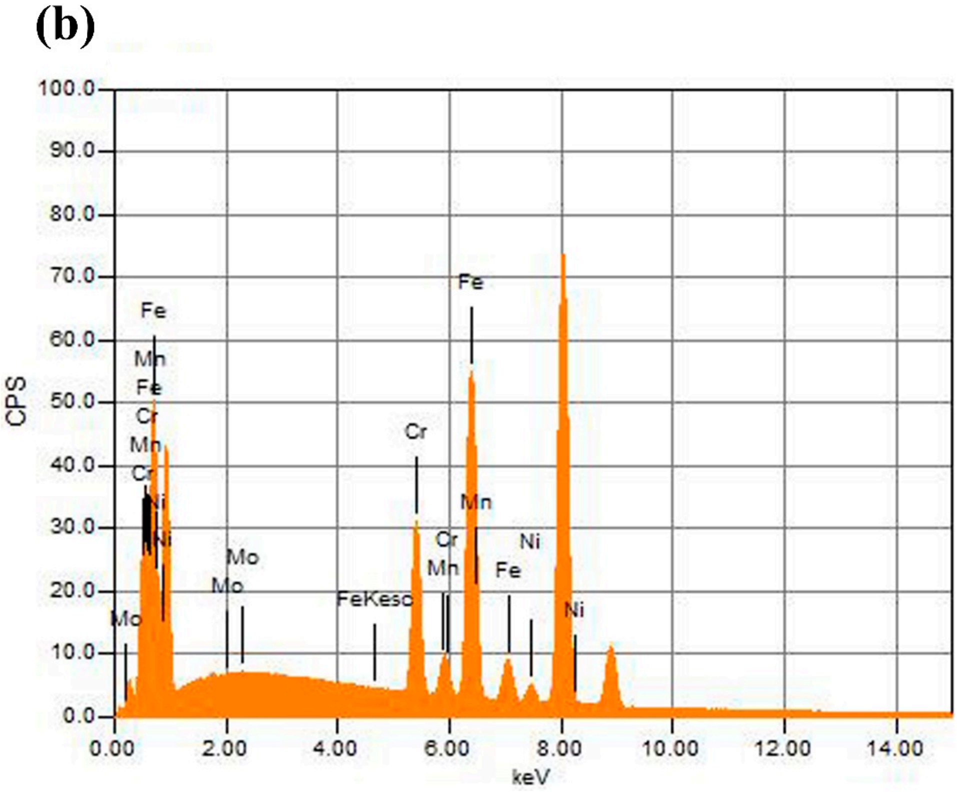

Figure S2. (**b**) EDS x-ray spectrum of the elements.

Surface topography analysis of SS films on SS 316L substrates:

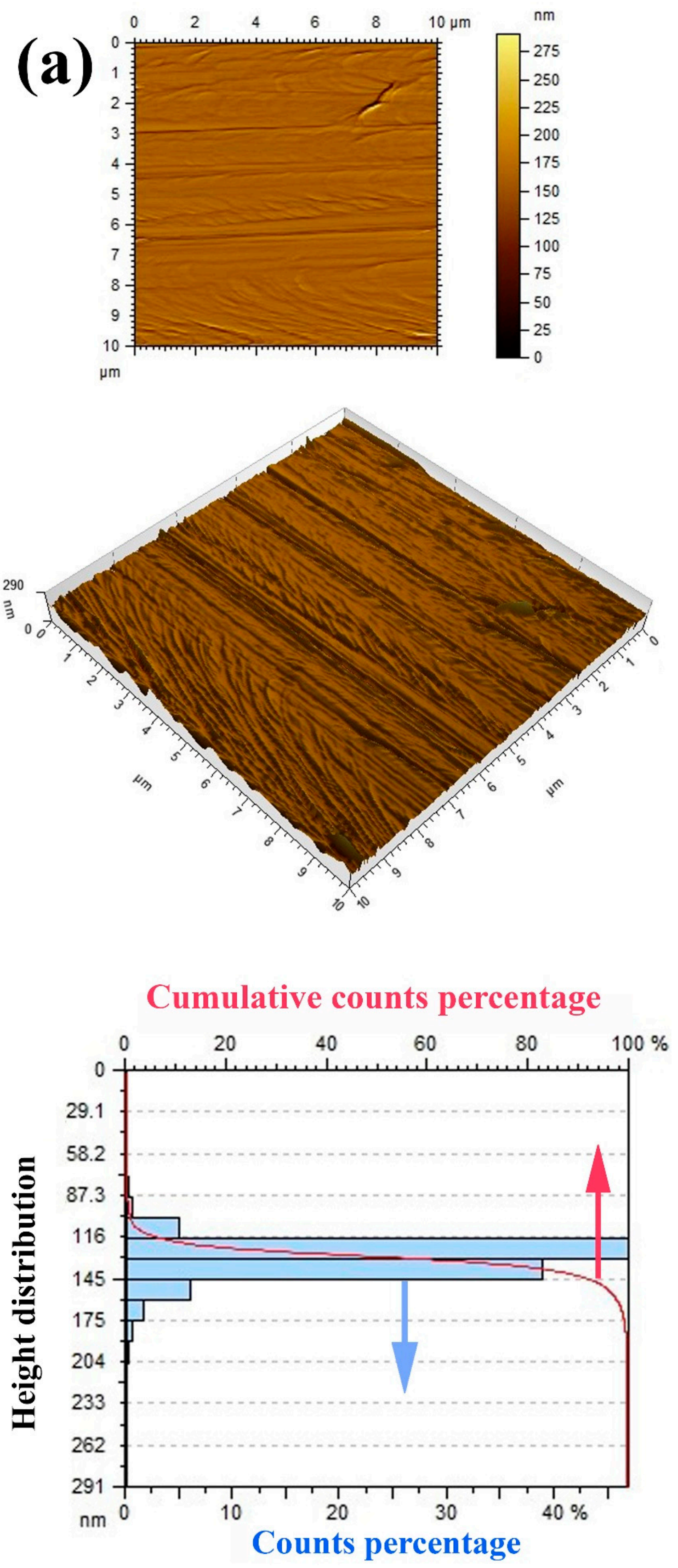

Figure S3. (**a**) AFM images and analysis of the uncoated substrate.

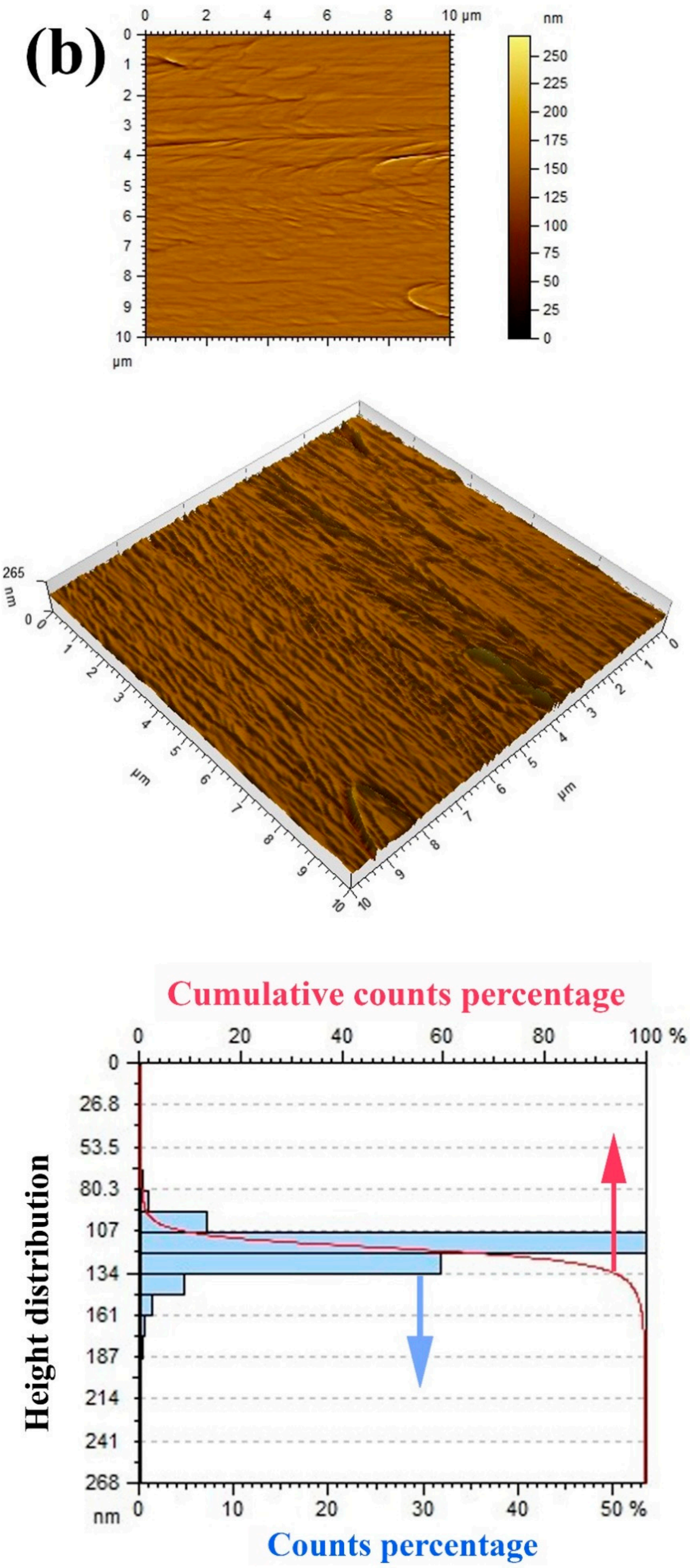

Figure S3. (**b**) AFM images and analysis of the 50 nm coated substrate.

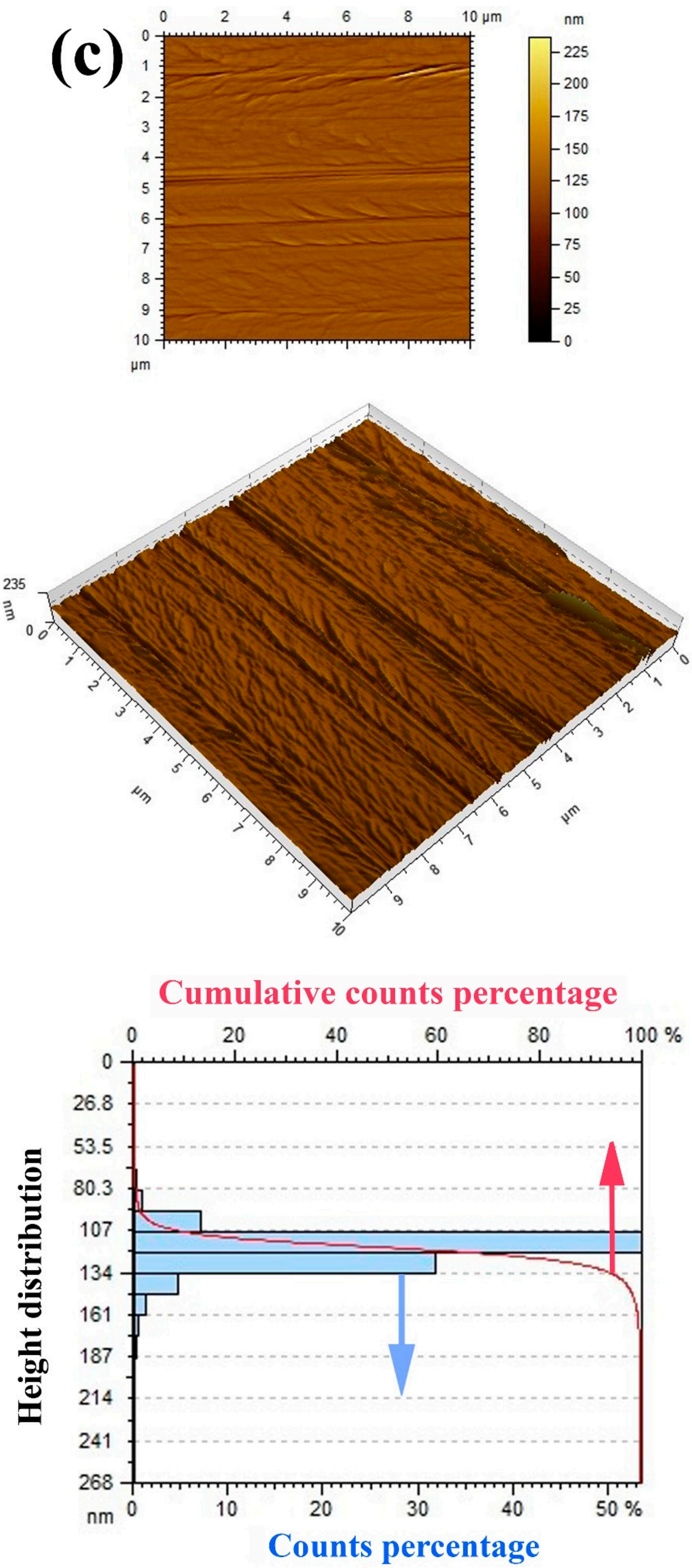

Figure S3. (**c**) AFM images and analysis of the 100 nm coated substrate.

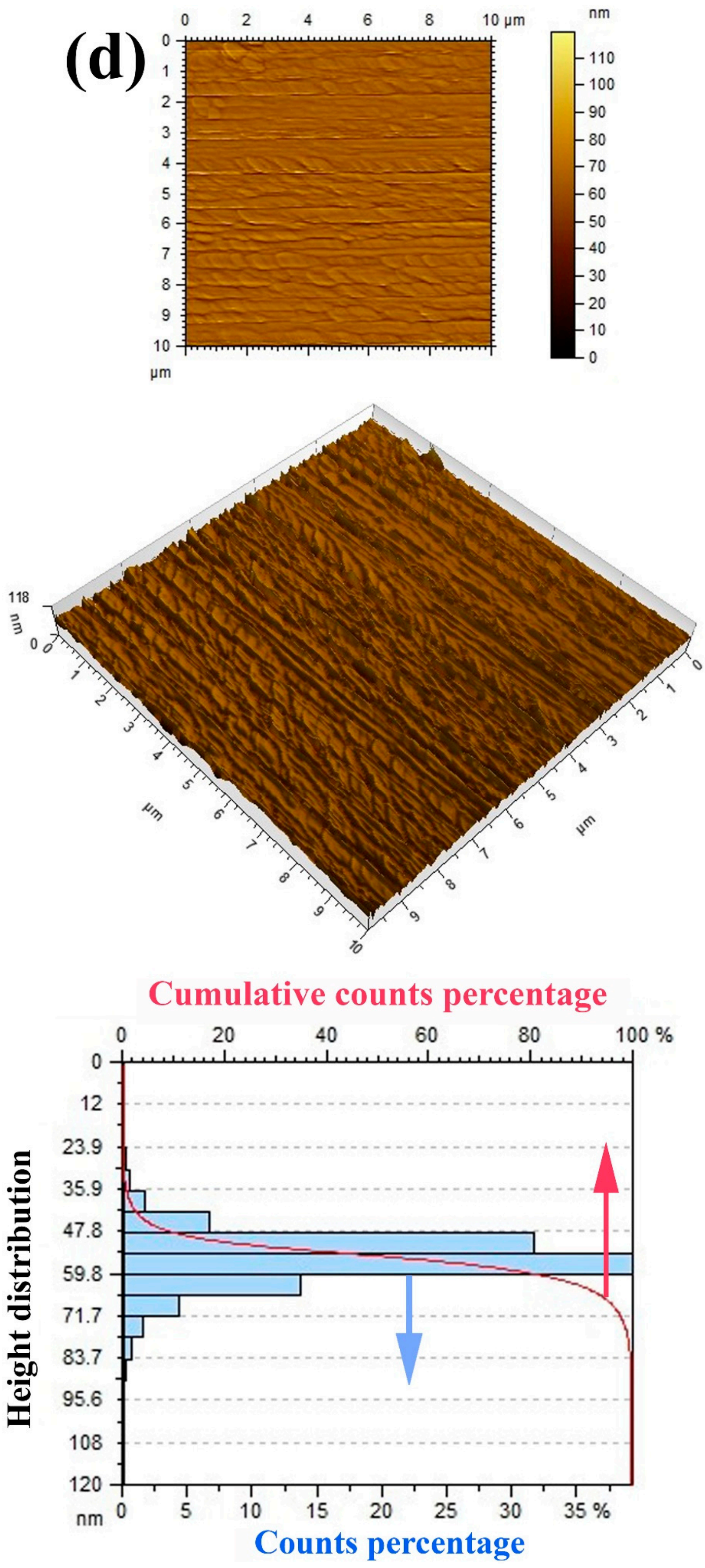

Figure S3. (**d**) AFM images and analysis of the 150 nm coated substrate.

Table S1. Contact angle measurements data.

DIW temperature (°C)	DIW pH value	Stainless steel substrate surface information	Highest CA (degree)	Lowest CA (degree)	ACA (degree)
20	4	Uncoated	126.5	122.5	124.9
30	4	Uncoated	120.6	119.4	119.8
40	4	Uncoated	106.5	106.2	106.3
50	4	Uncoated	110.3	110.1	110.2
60	4	Uncoated	112.7	112.1	112.3
20	7	Uncoated	132.8	130.9	131.7
30	7	Uncoated	122.5	119.5	120.6
40	7	Uncoated	115.8	115.6	115.7
50	7	Uncoated	115.2	114.5	114.8
60	7	Uncoated	115.3	114.8	115.1
20	9	Uncoated	117.8	116.7	117.4
30	9	Uncoated	115.7	114.8	115.3
40	9	Uncoated	108.3	108.0	108.1
50	9	Uncoated	112.7	112.1	112.3
60	9	Uncoated	107.4	106.8	107.1
20	4	50 nm SS film	121.0	117.8	119.5
30	4	50 nm SS film	110.8	110.4	110.6
40	4	50 nm SS film	104.2	103.8	104.0
50	4	50 nm SS film	110.1	108.7	109.3
60	4	50 nm SS film	110.7	110.2	110.4
20	7	50 nm SS film	131.3	130.1	130.9
30	7	50 nm SS film	116.7	114.8	115.8
40	7	50 nm SS film	105.1	104.9	105.0
50	7	50 nm SS film	104.6	104.1	104.4
60	7	50 nm SS film	101.3	100.8	101.1
20	9	50 nm SS film	109.6	108.7	109.2
30	9	50 nm SS film	106.6	106.4	106.5
40	9	50 nm SS film	108.7	107.4	108.1
50	9	50 nm SS film	100.6	99.0	99.7
60	9	50 nm SS film	105.0	104.9	104.9
20	4	100 nm SS film	117.4	115.8	116.7
30	4	100 nm SS film	104.0	103.5	103.8
40	4	100 nm SS film	103.8	103.3	103.5
50	4	100 nm SS film	99.0	98.1	98.5
60	4	100 nm SS film	102.1	101.3	101.7
20	7	100 nm SS film	128.3	128.1	128.2
30	7	100 nm SS film	109.6	108.8	109.3
40	7	100 nm SS film	103.4	102.8	103.0
50	7	100 nm SS film	103.3	102.7	102.9
60	7	100 nm SS film	100.6	99.0	99.9
20	9	100 nm SS film	103.3	103.0	103.2
30	9	100 nm SS film	103.4	103.2	103.3
40	9	100 nm SS film	106.2	105.1	105.7
50	9	100 nm SS film	99.7	98.1	98.8
60	9	100 nm SS film	98.4	97.5	98.1
20	4	150 nm SS film	111.0	110.6	110.9
30	4	150 nm SS film	99.8	98.1	99.0
40	4	150 nm SS film	102.8	102.0	102.5
50	4	150 nm SS film	94.4	92.1	93.1
60	4	150 nm SS film	88.7	88.4	88.5
20	7	150 nm SS film	122.6	121.0	122.0
30	7	150 nm SS film	106.7	106.4	106.6
40	7	150 nm SS film	102.9	102.5	102.7
50	7	150 nm SS film	95.8	95.1	95.5

60	7	150 nm SS film	95.5	94.7	95.2
20	9	150 nm SS film	95.0	94.2	94.5
30	9	150 nm SS film	101.3	100.9	101.0
40	9	150 nm SS film	103.4	102.9	103.2
50	9	150 nm SS film	97.5	96.2	97.0
60	9	150 nm SS film	97.4	96.7	97.0

© 2019 by the authors. Submitted for possible open access publication under the terms and conditions of the Creative Commons Attribution (CC BY) license (http://creativecommons.org/licenses/by/4.0/).
